# 

**Published:** 1902-06

**Authors:** 


### BOOKS RECEIVED.

The Drug Habits and Their Treatment.—A Clinical Summary of Some of the General Facts Recorded in Practice. By T. D. Crothers, M. D., Superintendent Walnut Lodge Hospital, Hartford, Conn.; Professor Diseases of the Brain and Nervous System, New York School of Clinical Medicine Pp., 96. Chicago: G. P. Englehard & Co. 1902. [Price, $1.00 net.]

The Perverts. By William Lee Howard, M. D. Duodecimo, pp. 388. New York: G. W. Dillingham Co. 1902. [Price, $1.50.]

Twenty-first Annual Report of the State Board of Health of New York. For the year ending December 31, 1900. Transmitted to the legislature February 18, 1901. With maps. Albany: James B. Lyon, State Printer. 1901.

A Manual of Surgical Treatment. By W. Watson Cheyne, M. B., F. R. C. S., F. R. S., Professor of Surgery in King's College, London; Surgeon to King's College Hospital, etc.; and F. F. Burghard, M. D. and M. S. (Lond.), F. R. C. S., Teacher of Practical Surgery in King's College, London; Surgeon to King's College Hospital, etc. In seven imperial 8vo volumes, with illustrations. Volume VI. 498 pages, with 124 illustrations. Philadelphia and New York: Lea Brothers & Co. 1902. [Price, cloth, $5.00 net.]

Ophthalmic Myology. A Systematic Treatise on the Ocular Muscles. By G. C. Savage, M. D., Professor of Ophthalmology in the Medical Department of Vanderbilt University; Author of "New Truths in Ophthalmology." Duodecimo, pp. 596; 61 illustrative cuts and 6 plates. Nashville, Tenn: Published by the author. 1902.

A Practical Manual of Bacteriology for Students and Physicians. By A. C. Abbott, M. D, Professor of Hygiene, University of Pennsylvania. New (6th) edition, revised and enlarged. In one 12mo volume of 636 pages, with 111 illustrations, 26 colored. Philadelphia and New York: Lea Brothers & Co. 1902. [Price, cloth, $2.75 net.]

The Medical Treatment of Gall-Stones. By J. H. Keay, M. A., M. D. Duodecimo, pp. 133. Philadelphia: P. Blakiston's Son & Co. 1902. [Price, $1.25.]

The Practical Medicine Series of Year Books. Ten volumes. Issued monthly under the general editorial charge of Gustavus P. Head, M. D., Professor of Laryngology and Rhinology in the Chicago Post-Graduate Medical School. Volume V. Obstetrics. Edited by Reuben Peterson, A. B., M. D., Professor of Obstetrics and Gynecology in the University of Michigan; and Henry F. Lewis, A. B., M. D., Instructor in Obstetrics and Gynecology in Rush Medical College. Duodecimo, pp. 233. Illustrated. Chicago: The Year Book Publishers. 1902. [Price, $1.25.]

The Operations of Surgery. By W. H. A. Jacobson, M.Ch., Oxon, F.R.C.S., Surgeon to Guy's Hospital; Consulting Surgeon Royal Hospital for Children and Women; Member Court of Examiners, Royal College of Surgeons, etc.; and F. J. Stewart, M. S., London, F. R. C. S., Assistant Surgeon, Guy's Hospital and to the Hospital for Sick Children; Surgeon-in-Charge of the Throat Department, Guy's Hospital. Fourth edition, revised, enlarged and improved. 550 illustrations. Two volumes. Volume I.' Operations on the Upper Extremity; Operations on the Head and Neck; Operations on the Thorax. Volume II. Operations on the Abdomen; Operations on the Lower Extremity; Operations on the Vertebral Column. Philadelphia: P. Blakiston's Son & Co. 1902. [Price, cloth, $10.00; sheep, $12.00 net.]

Proceedings of the American Medico-Psychological Association, at the fifty-seventh annual meeting, held at Milwaukee, Wis., June 11–14, 1901. C. B. Burr, M. D., Secretary. Published by the Association. 1901.

Quain's Dictionary of Medicine. By various writers. Edited by H. Montague Murray, M. D., F. R. C. P., Joint Lecturer on Medicine, Charing Cross Medical School, and Physician to Out-patients, Charing Cross Hospital; Senior Physician to the Victoria Hospital for Children, Chelsea, and to the Foundling Hospital. Assisted by John Harold, M. B., B. Ch., B. A. O., Physician to St. John's and St. Elizabeth's Hospital, and Demonstrator of Medicine at Charing Cross Medical School; and W. Cecil Bosanquet, M. A., M. D., M. R. C. P., Physician to Out-patients, Victoria Hospital for Children, Chelsea, and Pathologist to Charing Cross Hospital; Late Fellow of New College, Oxford. Third edition, largely rewritten and revised throughout. With 14 colored plates and numerous other illustrations. New York: D. Appleton & Co. 1902. [Price, half-morocco, $10.00.]

The Surgery of the Rectum. By Charles B. Kelsey, A. M., M. D., Late Professor of Pelvic and Abdominal Surgery at the New York Post-Graduate Hospital, and Professor of Rectal Surgery at the University of Vermont. Octavo, pp. 411. Sixth edition. Illustrated by 215 engravings. New York: William Wood & Co. 1902. [Price, $3.00.]

International Clinics. A Quarterly of Clinical Lectures and Especially Prepared Articles on Medicine, Neurology, Surgery, Therapeutics, Obstetrics, Pediatrics, Pathology, Dermatology, Diseases of the Eye, Ear, Nose and Throat, etc. Edited by Henry W. Cattell, A. M., M. D., of Philadelphia, with the collaboration of John B. Murphy, M. D., of Chicago; Alexander D. Blackader, M. D., of Montreal; H. C. Wood, M. D., of Philadelphia; T. M. Rotch, M. D., of Boston; E. Landolt, M. D., of Paris; Thomas G. Morton, M. D., of Philadelphia; J. W. Ballantyne, M. D., of Edinburgh, and John Harold, M. D., of London Volume I. Twelfth series 1902 84 illustrations, with 3 colored plates. Philadelphia: J. B. Lippincott Co. [Price, cloth, $2.00 each; half-leather, $2.25 each.]

